# Account of Medical Education in Edinburgh, with Remarks

**Published:** 1802-01

**Authors:** 


### *Account of Medical Education in Edinburgh, with Remarks.*

To the Editors of the Medical and Physical Journal.

Gentlemen,

I Took the liberty, fome time ago, of fending you an account of the manner in which Medical Education is conducted in Edinburgh, and now beg the favor of you to infert the mifcellaneous remarks on the fame fubject, which I then promifed you.

The opportunities afforded at this Univerfity of profecuting the ftudy of medicine are not confined to the College. Private lectures are alfo given in Anatomy, Surgery, Phyfiology, and Chemiftry, by men of merit and eminence, which are of confiderable advantage to the ftudent, particularly in the ftudy of Anatomy. This fcience is the only one in which Edinburgh has not arrived at a confiderable degree of eminence, and it is in a great meafure owing to the difficulty there has been in procuring a fufficient number of fubjects for private diffection. The exertions of the private teachers have, however, in fome meafure, done away this difadvantage, and the more frequent opportunities of profecuting practical Anatomy which are now enjoyed, muft neceffarily be of material fervice to the Univerfity. Anatomy cannot be learnt from lectures alone, for however perfpicuous the demonftrations may be which accompany them, the ideas which they produce muft remain in fome meafure indiftinct and imperfect without the aid of diffection, and the more fo when they are given to a very large number of ftudents at one time, as is the cafe in Edinburgh. It is of extreme importance in the ftudy of any fcience, that the firft rudiments fhould be correct and diftinct. Imperfect ideas are eafily acquired, but they greatly retard the after progrefs, and are fometimes never entirely removed. If there is, therefore, a defideratum

ſideratum in the Medical Education of Edinburgh, it is, that more opportunities ſhould be afforded to the ſtudent of proſecuting the ſtudy of Anatomy. There are certainly many obſtacles in the way of procuring ſubjects ſufficient to enable the greater number of medical ſtudents to cultivate practical Anatomy, and it is not improbable that the popular prejudices againſt diſſection, and the more limited opportunities of obtaining bodies in Edinburgh than in London, will make it always inferior to the latter as an Anatomical School. The ſocieties which are inſtituted in Edinburgh for diſcuſſing Medicine and Medical Philoſophy have a conſiderable tendency to encourage the deſirable habits of ſtudy and inveſtigation. It muſt be admitted, however, that they have their diſadvantages; for to young men, who have juſt aſſumed the right of thinking for themſelves, without having obtained that information which is neceſſary as the foundation of their opinions, they frequently hold out a dangerous and ſuccesſful allurement, which diverts them from the more ſubſtantial parts of their ſtudies, renders them diſputatious, and encourages habits of ſelf-complacency and conceit. It is, perhaps, a good deal owing to the effects of ſocieties in general, that the ſtudents of Edinburgh obtained the character from Dr. Franklin of being, with lawyers, particularly fond of diſpute. Upon the whole, however, it appears, that the more reſpectable of the medical ſocieties are conducive to profeſſional improvement, if they are only regarded as ſubſervient to the other parts of education, if they are not attended too early, and if the ſtudent does not, as is too frequently the caſe, take a part in the diſcuſſion beyond his information.

The *Royal Medical*, and the *Royal Phyſical*, are the two principal Societies devoted to ſubjects connected with medicine. The former ſtands higheſt in point of general reſpectability and importance, and has, in the liſt of its members, many of the moſt diſtinguiſhed medical characters in Europe. It was here, as Dr. Gregory remarks in his lectures, that the Boerhaavian doctrines were firſt freely diſcuſſed, and ſuch objections urged againſt their ſolidity, as gradually led to their abandonment. The members aſſume the full right of freedom of diſcuſſion on any opinions, by whatever authorities they may be ſupported, and are extremely jealous of the ſmalleſt endeavour, on the part of the profeſſors, to controul them. During the time that the celebrated doctrines of Brown firſt began to be canvaſſed, the warmth of debate was carried on ſo far, as to render it neceſſary to paſs a law (which ſtill exiſts) to enact, that any gentleman who ſhould challenge another, on account of any thing which might take place during debate, ſhould be expelled the ſociety.

The Royal Medical Society was inſtituted in the year 1737, and obtained a charter of corporation in 1778. It is governed by four Preſidents, and by committees choſen annually from the members by ballot. The meetings are held once a week, and laſt from 7 in the evening till 12. An eſſay on a medical caſe, and a diſſertation on ſome ſubject connected with medicine, (which are written by the ordinary members in rotation, and are termed a ſet of papers) are diſcuſſed every evening, and not more than two ſets of thoſe papers are expected from any gentleman during the time of his being in the ſociety. They are circulated among the members before diſcuſſion, and are copied into proper books, which are open to their inſpection in the library. Fines are exacted for abſence either during the whole or part of the evening, and theſe are ſo conſiderable as to enforce a pretty punctual attendance. The admiſſion fee is five guineas; one guinea is paid in the beginning of the ſecond year of attendance, and half a guinea in the beginning of the third, after which no further ſum is required. A member, after having written two ſets of papers, and completed two ſeſſions in the ſociety, becomes extraordinary, and is then freed from fines or contributions.

This ſociety is poſſeſſed of a very valuable library and philoſophical apparatus, which are under excellent regulation. Periodical publications, and new works lie a certain time upon the library table, for the uſe of the members, before they are put into the body of the library.

There are ſeveral circumſtances which have tended to eſtabliſh the reputation of Edinburgh, and to make it a very general reſort for medical ſtudents. Two or three of thoſe it may not be improper to mention.

The celebrity of its profeſſors, without doubt, brought it firſt into notice, and on this, its eſtimation in the eyes of Europe muſt ſtill depend. The eſtabliſhment of profeſſors in Edinburgh has a powerful tendency to preſerve that zeal and activity in the proſecution of the ſubjects which they teach, ſo neceſſary to the advancement of ſcience and the increaſe of perſonal reputation. The ſalaries are very ſmall, their emoluments muſt therefore principally depend upon the manner in which their lectures are attended, and this again on the eſtimation in which they are held. In Edinburgh, too, the practice of a phyſician is very much limited. Lecturing is not, therefore, as is often the caſe in London, employed as an introduction merely to public notice, but is regarded as a profeſſion to which which a man feels it his intereft to direct all his abilities and attention.

Without having the moft diftant intention of making any application on the fubject, I may be allowed to obferve, that a more refpectable phalanx of medical men could fcarcely be brought together, than filled the profeffional chairs of this Univerfity a few years ago. Any one converfant with its hiftory, muft know that I allude to the period at which Dr. Cullen, Dr. Black, Dr. Gregory, Dr. Hope, Dr. Monro, and Dr. Home, were all of them at one time engaged in teaching the different branches of medical fcience. It is extremely difficult to point out that mode of electing profeffors, which is moft likely to enfure their being properly qualified for their office.

The members of a town council, do not appear to be the men beft adapted for this purpofe, and yet it is with them that the nomination refts. Hitherto, there has been little ground of complaint; it may eafily however be feen, that the utmoft circumfpection is neceffary to preferve the refpectability of the profeffional chairs in the eyes of Europe, and that the fyftem which has been adopted of late years, of appointing the fon as the fucceffor or colleague of his father, if carried to far, will be highly injurious to the interefts of the Univerfity.

The habits of life, both of the ftudents and the inhabitants in general of Edinburgh, are very favourable to the fteady purfuit of fcientific knowledge. The ftudents are not men of fortune, who come to the Univerfity for the purpofe of *lounging away* two or three years for the fake of form or fafhion, but are fuch as depend for their fuccefs, on their perfeverance, affiduity, and attention in the purfuits to which they are devoted. Enquiry is encouraged by the profeffors, excited by the eafy intercourfe which takes place among the ftudents, and readily gratified by the facility with which books can be procured, whether from the College Library or that of the Royal Medical Society. The defire of diftinction among themfelves, and the wifh to come off with credit on an application for a degree, have alfo a very confiderable and falutary influence upon the ftudents.

The inhabitants of Edinburgh are, in general, diftinguifhed for information and propriety of behaviour, which may be attributed to a variety of caufes. Learned and literary men, particularly thofe connected with the college, are held in great eftimation, and are received with cordiality into the higheft circles. By far the moft numerous clafs of the refpectable inhabitants are in the profeffions; and commerce, which fometimes fuddenly raifes ignorant and ill educated men to affluence, has fcarcely any place in the Scottifh capital. Thefe circumftances

ſtances cannot be without ſome effect upon the minds of young men who go thither for the purpoſe of improvement.

The Lectures themſelves are more comprehenſive than thoſe, which, as far as I know, are delivered in any other Univerſity in Europe; and the Infirmary, as I mentioned in my former paper, is under regulations, which render it productive of much advantage to the ſtudent.

The eſtimation in which Edinburgh is held as a ſchool of medicine, is ſufficiently marked by the general reſort of ſtudents to it from every part of Europe. Few medical men, who can boaſt of a regular courſe of ſtudy, have not enjoyed the advantages of this ſchool; and by much the greater number of the moſt reſpectable phyſicians of this iſland, have graduated in Edinburgh. It may hardly be thought neceſſary to make any obſervations on the reſpectability of Edinburgh degrees, after the detail which I have given, and the general eſtimation in which they are held with the public; I think it proper however to obſerve, that, to the diſgrace of an honourable profeſſion, and of an enlightened nation, which ought, on account of its uſefulneſs, to preſerve its reſpectability, the right of practiſing medicine is at this time to be purchaſed at a Scotch Univerſity without ſtudy, attendance, or examination; ſimply by a certificate of fitneſs from two phyſicians, who, it is to be feared, too often ſign it from courteſy, without being adequately informed of the education or acquirements of the perſon whom they recommend. It ought therefore to be recollected by thoſe who, from this circumſtance, depreciate the value of Scotch degrees, that it is unfair and uncandid to claſs the Univerſity of St. Andrew's with that of Edinburgh, where the *eligibility to examination for a degree* can only be obtained by three years regular ſtudy, and where the examination itſelf is well adapted to diſcover how far the ſtudent has availed himſelf of the opportunities he has enjoyed.

It ſeems indeed highly prepoſterous, that a qualification to practiſe a liberal profeſſion, like that of medicine, which has not only its general character for learning to ſupport, but which alſo requires no ſmall ſhare of ſtudy and application to become acquainted with its principles, ſhould be able to be purchaſed by any one, who chuſes to be at the expence of it; for though it be admitted, that the certificate of qualification be in ſome meaſure a check upon the introduction of any improper perſons to the honours of medicine, yet is it to be imagined, that medical men cannot occaſionally be found, who will ſo far forget their own honour and the honour of their profeſſion, as to recommend men very ill adapted to ſupport its credit and reſpectability? In the profeſſions of Law and Divinity, nity, it is required that a man ſhould ſpend a certain time in the ſtudy of his profeſſion before he is admitted to practiſe it; or, at leaſt, that he ſhould be of ſo long a ſtanding as to give him a full opportunity of having turned his attention to it; and even then a certificate of fitneſs is required previous to admiſſion. If ſuch be proper or neceſſary in the profeſſions which I have mentioned, is it not doubly ſo in that of medicine, where the health or the life of individuals depend upon the knowledge and judgement of one man, and where there is often no time for looking into ſources of information, or endeavouring to ſupply a deficient knowledge of the ſubject? The evil is ſerious; and it is the duty of every good man, who wiſhes well to the profeſſion of medicine and to the public, to do his endeavours to remedy it. For though ſome individual inſtances may occur, in which a man may be poſſeſſed of every qualification neceſſary for a phyſician, and would be much inconvenienced by not having a medical degree without the loſs of time occurred by an attendance at an Univerſity for the purpoſe of obtaining it, yet the poſſibility of ſuch an occurrence ought not to prevent any meaſure of univerſal public and profeſſional advantages. It would not be thought ſufficient for a perſon in a ſubordinate part of the law, who had been all his life engaged in the practice of his profeſſion, to ſay, that he expected to be at once admitted to the bar, becauſe he conceived himſelf qualified to practiſe as a counſel. He would be expected to purſue the ſame courſe, as if he had been juſt entering upon the ſtudy of the law, however inconvenient might be the delay. And it does not appear particularly unreaſonable, that when a profeſſion is either entered upon or changed, a ſacrifice of two or three years ſhould be made, in order to obtain a qualification to practiſe it, though it may occaſionally be attended with ſome perſonal inconvenience.

The Royal College of Phyſicians of London ſeem perfectly convinced of the propriety of requiring a certain academical attendance from every one, previous to examination for the liberty of practiſing in the metropolis; when they make it neceſſary for ſuch as preſent themſelves, to produce documents of having ſtudied two years at the ſame univerſity where they graduated. This bye law appears to be founded upon the moſt judicious principles, and does credit to the judgment of that learned body. The latter part of it, is clearly with a view to prevent thoſe from applying for admiſſion as licentiates, who were rejected on their examination for a degree at the Univerſity where they were educated, and afterwards procured one by purchaſe.

The Royal College of Phyſicians of Edinburgh have a defect, fect in their charter, which prevents their being able to adopt the fame falutary regulations with that of London, on the mode of admitting licentiates or fellows. By their charter it is enacted,* that not only graduates of Edinburgh, but of any other Scotch Univerfity, fhall be admitted by right a licentiate of the college without examination; and by the conftitution of the college, a licentiate, after remaining twelve months in that probationary ftate, is admitted a fellow as a matter of courfe.

That Edinburgh graduates fhould be admitted without examination into the College of Phyficians, is not improper, becaufe the heads of this college are members of the faculty of Medicine of the Univerfity, and the perfons who on examination thought him duly qualified to practife medicine. But, that any perfon who has purchafed a St. Andrew's degree, fhould be able to claim the fame privilege, completely does away any idea of honour from a connection with that body. The abfurdity of the claufe above mentioned, more clearly appears from a cafe which is not only poffible, but may at any time occur. A ftudent may be thought unqualified for the honour of a degree in Edinburgh, and may be rejected. He may afterwards procure a degree from St. Andrew's, and infift upon admiffion into the College of Phyficians of Edinburgh, though he will be there affociated with the very men who thought him unworthy of a degree from the Univerfity. Thus it appears, that the degree of Doctor from the Univerfity is more honourable than that of Fellow of the Royal College of Phyficians, a circumftance which, I believe, is far from being generally known, though it may be very fairly inferred from the refpectable evidence of Dr. Gregory, who made the obfervation to which I alluded above, when actually prefident of the College.

On a comparifon of the advantages afforded by Edinburgh and the Englifh Univerfities, in the ftudy of medicine, there can be no hefitation in preferring the former. As an argument in favour of Oxford and Cambridge, much ftrefs has been laid upon the length of time which it is neceffary to be connected with thofe Univerfities, before the degree of Doctor can be obtained. But in this point of view, the balance muft be on the fide of Edinburgh, for a great part of the attendance at the Englifh Univerfities is merely nominal, half terms only being fufficient before taking the degree of Batchelor of Medicine; and from that time until the period at which the degree of Doctor is conferred, no attendance is neceffary at all.

Does

* Vide Dr. Gregory's Letter to the Managers of the Royal Infirmary.

Does the period of abfolute attendance, therefore, which is neceffary previous to taking a Doctor's degree, predominate in Edinburgh, or in the Englifh Univerfities? Much of the advantages derived from a place of education, neceffarily depends upon the time and punctuality of attendance; and this applies more ftrongly in medicine, than perhaps any other profeffion. When we therefore confider that the period of *actual attendance* in Edinburgh is equal to that which is requifite at either of the Englifh Univerfities; and when we alfo recollect, that during the whole of that attendance, the time is occupied in the proper bufinefs of the profeffion, little doubt can exift on the comparative merits of thofe feminaries as fchools of medicine. It is ufual, and indeed neceffary, that gentlemen educated to Phyfick at either of the Englifh Univerfities, fhould profecute the ftudy of their profeffion elfewhere; but is it not a circumftance of a moft fingular nature, that the only advantage in medicine from thofe illuftrious feminaries, is the obtaining a degree? and that the previous acquirements which are neceffary to qualify a man to practife with honour to himfelf, or advantage to the world, muft be made in another place?

I have thus, Gentlemen, completed the mifcellaneous Obfervations on Medical Education in Edinburgh, which I had in view, and fhould be gratified by their affording any degree of intereft to you or your readers.

*London, Dec. 6, 1801.* Y.

